# Advances in Fruit and Vegetable Quality, Bioactive Compounds, and Nutritional Value: 2nd Edition

**DOI:** 10.3390/foods14152665

**Published:** 2025-07-29

**Authors:** Andrea Gómez-Zavaglia, Lucía Cassani

**Affiliations:** 1 Center for Research and Development in Food Science and Technology (CIDCA), CCT-CONICET, RA1900 La Plata, Argentina; angoza@qui.uc.pt; 2 Departamento de Química Analítica y Alimentaria, Facultad de Ciencias, Universidade de Vigo, 32004 Ourense, Spain; 3 Instituto de Agroecoloxía e Alimentación (IAA), Universidade de Vigo, Campus Auga, 32004 Ourense, Spain

## 1. Introduction

In recent years, substantial progress has been made in characterizing the quality of fruits and vegetables, identifying bioactive compounds, and developing novel food matrices with enhanced nutritional value. Advances in postharvest handling, food processing technologies, and analytical methods have enabled a more in-depth understanding of the biochemical and functional changes that occur throughout the production chain [1]. At the same time, there is a growing emphasis on the valorization of agro-industrial co-products and the shift toward sustainable food systems that align with both environmental and public health goals, issues that have direct implications for our daily lives, business models, and environmental sustainability [2].

The importance of these issues underscores the urgent need to rethink and redesign food value chains in line with global frameworks such as the United Nations Sustainable Development Goals (SDGs) and the European Union’s Green Deal. The SDGs, particularly Goals 2 (Zero Hunger), 3 (Good Health and Well-being), 12 (Responsible Consumption and Production), and 13 (Climate Action), promote inclusive sustainable practices across all stages of food systems, aiming to end hunger, improve nutrition, and fight against climate change. Similarly, the European Green Deal, an ambitious roadmap toward climate neutrality by 2050, prioritizes the transformation of food systems through its “Farm to Fork” strategy, which aims to reduce the environmental footprint of food production, improve food security, and ensure access to healthy sustainable diets [3,4]. In the Ibero-American context, agri-food systems have been identified as a strategic pillar within the Ibero-American Innovation Missions, a cross-sectoral initiative intended to address major regional challenges through science, technology, and innovation [5]. This initiative gains further momentum as we approach the 2026 Ibero-American Summit of Heads of State and Government in Madrid. The importance of food systems in these high-level discussions reflects their broad impact on sustainability, public health, economic resilience, and regional equity. All these policy efforts converge around shared objectives, namely reducing food loss and waste, promoting sustainable production and consumption patterns, and fostering inclusive circular bioeconomies.

This evolving political and scientific landscape supports and motivates further research aimed at closing critical knowledge gaps. For instance, many fruit and vegetable species remain underexplored in terms of their bioactive profiles and potential health benefits. Further investigation is also needed into the stability of functional compounds during storage and processing, the optimization of agricultural practices to enhance quality, and the incorporation of food safety considerations into innovative product formulations. Industrial-scale recovery and utilization of functional ingredients from co-products, especially within a circular economy model, also remain major technical and logistical challenges.

All these aspects strongly support the publication of a second edition of this Special Issue, which includes original contributions that directly address the existing knowledge gaps, presenting novel approaches for improving the quality and functionality of fruit- and vegetable-based foods. We hope the research showcased in this second edition not only enriches ongoing scientific discussions but also provides valuable evidence to inform policy decisions that support more sustainable, equitable, and innovation-driven agri-food systems.

## 2. Overview of the Published Articles

The second edition of this Special Issue brings together a diverse collection of ten contributions, with nine original research articles and one review. Most of the studies focus on evaluating the quality parameters of fruit- and vegetable-based products, with particular emphasis on their bioactive properties, biological activities, and changes during storage. Some works also address food safety concerns and the characterization of bioactive profiles. In the following section, we summarize the main objectives and key findings of each contribution to encourage readers to explore them in detail.

As the first contribution to this Special Issue, Gubitosa et al. employed callus culture techniques on ripe *Annurca* apple pulp (*Malus pumila* cv. Miller) under two light conditions (darkness and an 18 h photoperiod) to enhance the production of secondary metabolites. This technique involves cultivating aggregates of irregular undifferentiated plant cells capable of producing metabolites similar to those of the parent tissue. These cells can modulate their metabolic pathways and adapt their growth in response to environmental stimuli. Notably, the authors reported the production of substantial quantities of triterpenic compounds not previously identified in apple pulp. Furthermore, the callus extracts exhibited anti-inflammatory and genoprotective properties, although their antioxidant activity was comparatively lower.

The article of Vincenzo et al., the second contribution to this Special Issue, studies multiple quality-related parameters of dried kiwifruit slices (40 and 55 °C, for 30 and 25 h, respectively) stored for 120 days at 25 °C. The parameters studied were nutritional (total phenolic content, flavonoids, ascorbic acid, organic acids), functional (antioxidant activity), physicochemical (pH, water activity, titratable acidity, color), and textural and sensory properties (hardness, chewiness, aroma intensity). The authors observed that some quality parameters remained unchanged over storage time.

In the third contribution, Cásedas et al. studied the cytoprotective and neuroprotective properties of a solvent-free hydrophilic extract of black mulberry (*Morus nigra* L.), rich in polyphenols. The study shed light into its antioxidant, antiradical, and enzymatic mechanisms of action. The results confirmed the extract’s neuroprotective potential, highlighting its capacity to scavenge free radicals, reduce reactive oxygen species (ROS) production, and moderately inhibit monoamine oxidase A (MAO-A). Furthermore, the polyphenol-rich extract mitigated β-amyloid-induced toxicity in *Caenorhabditis elegans*. Overall, the findings support the potential of *Morus nigra* as a functional food containing bioactive compounds that may contribute to the prevention of neurodegenerative diseases.

The fourth contribution, a review article by Golowczyc and Gomez-Zavaglia, studies the negative consequences of antibiotic use in livestock farming and emphasizes the need to address the challenges and safety concerns associated with extracting value-added ingredients from fruit and vegetable co-products at an industrial scale. The review also explores current trends aimed at reducing antibiotic use in animal production, with a particular focus on the Latin American context. In addition, it discusses the potential of incorporating these value-added ingredients into animal feed formulations. The authors suggested that using fruit and vegetable co-products as feed additives offers a dual advantage: it reduces the environmental footprint of food waste and supports the sustainability of livestock production systems. By incorporating bioactive inhibitory compounds into animal diets, producers can decrease their reliance on conventional antibiotics, thereby tackling public health issues linked to antibiotic resistance while improving farm efficiency and productivity. This forward-thinking strategy not only aids food waste management but also promotes a more sustainable and health-oriented approach to animal agriculture.

The fifth contribution, by Łata et al., evaluated 21 fruit genotypes, several of which were analyzed for the first time, using multiple methods to determine phenolic content and total antioxidant capacity, along with HPLC analysis to quantify total ascorbate in freshly harvested fruits. Among the studied genotypes, *Chaenomeles × californica*, *Actinidia kolomikta*, *Mespilus germanica*, and *Sorboaronia fallax* emerged as particularly promising candidates for further investigation due to their elevated levels of phenolics, ascorbate, and antioxidant potential. The study highlights the need for more comprehensive research to assess discrepancies among total phenolic content measurement techniques and to deepen the chemical characterization of these underutilized fruits.

The article by Modica et al., presented as the sixth contribution, explores the qualitative characteristics of several blood orange cultivars grown across three different environments in Spain and Italy. The authors measured the accumulation of primary and secondary metabolites, including bioactive compounds, and evaluated key fruit quality traits at harvest. Overall, the cultivars Moro and Sanguinelli were confirmed as highly suitable for juice production due to their intense pigmentation, while Tarocco lines demonstrated greater suitability for fresh consumption. The cultivation environment significantly influenced juice color, with Moro and T. Ippolito displaying the highest levels of pigmentation. Moreover, the geographical location affected the accumulation patterns of both primary and secondary metabolites in the Tarocco cultivars, as well as their antioxidant potential. Principal component analysis revealed three distinct clusters: two overlapping clusters corresponding to the Spanish sites and a clearly separated third cluster representing the genotypes cultivated in Italy.

The seventh contribution, by De Bruno et al., follows a similar approach to that of Modica et al. (2024), focusing on the composition of bioactive compounds and their antioxidant activity in various fig accessions cultivated in the Calabria region of Italy. The analysis revealed notable differences in phenolic profiles among the samples, as well as between the flesh and the skin of the fruits. In general, the skin contained significantly higher concentrations of bioactive compounds than the flesh. Accessions with dark-colored skin exhibited greater total antioxidant capacity compared to those with lighter skin. Notably, the CS147 accession (*Black Biferous*) demonstrated the highest antioxidant activity in the DPPH assay, both in the flesh and skin. Five phenolic compounds were identified, representing different chemical classes: phenolic acids (chlorogenic acid), flavonoids (catechin and epicatechin), and flavonols (quercetin and rutin).

The study by Brandl et al., presented as the eighth contribution, addresses food safety concerns related to fresh beverages made from blended raw fruits and vegetables, focusing on the behavior of *Escherichia coli* O157:H7 in leafy greens such as spinach and various lettuces. The authors found that *E. coli* proliferation was not linked to the sugar content but was negatively associated with hydrogen peroxide (H_2_O_2_) levels in leaf tissue. Higher H_2_O_2_ concentrations, especially in older leaves, appeared to inhibit bacterial growth. These findings highlight the importance of maintaining bioactive antimicrobial compounds such as reactive oxygen species (ROS) in fresh produce, suggesting that microbial safety depends more on these natural defenses than on sugar levels. The study recommends preserving ROS during processing to limit pathogen growth under temperature abuse conditions.

The ninth contribution, by Polyiam et al., evaluated the bioactive compounds, antioxidant capacity, and neuroprotective properties of a plant-based mixture of mung bean and mulberry fruit powder (MMP) in a 1:3 ratio. The formulation showed synergistic inhibition of acetylcholinesterase (AChE) and maintained over 50% stability of phytochemicals and bioactivities under both real-time (6 months) and accelerated (8 weeks) storage conditions. Optimal storage was at 4 °C or below, preserving the antioxidant and neuroprotective effects, including inhibition of DPPH, FRAP, AChE, MAO, MAO-A, and GABA-T, with IC_50_ values ranging between 4.10 and 6.69 mg/mL. The authors suggest MMP has promising potential as a stable plant-based neuroprotective food supplement for industrial application.

The tenth and final contribution, by Prisacaru et al., also addresses shelf-life assessment, focusing on the microbiological and physicochemical stability of five commercial fruit jams (apricot, sour cherry, white cherry, raspberry, and strawberry) during storage at 2–4 °C and 20 °C. While refrigeration helped slow down changes, both storage conditions led to measurable changes in pH, titratable acidity, moisture, total sugars, viscosity, and color, especially under room temperature. Microbial growth (yeasts, molds, and aerobic mesophilic bacteria) increased over time, influenced by temperature, storage duration, fruit type, and production conditions. The study highlights the importance of refrigeration after opening and recommends consuming jams within 14 days to ensure safety and quality.

As we look ahead, future research should continue to explore underutilized plant species and their potential as sources of novel bioactive compounds. Greater efforts are also needed to scale up sustainable technologies for ingredient extraction and food processing, ensuring functionality, safety, and environmental efficiency across the value chain. The integration of advanced omics technologies, green extraction methods, and life cycle assessment tools can further enhance our understanding of food system sustainability. We hope this Special Issue serves not only as a scientific contribution but also as a call to action for researchers, industry stakeholders, and policymakers to collaboratively shape more resilient, health-promoting, and environmentally responsible agri-food systems.

## 3. Conclusions

This second edition of the Special Issue provides valuable insights into the evolving landscape of fruit- and vegetable-based products, highlighting advancements in both bioactive compound research and food quality and safety assessment. Across the ten contributions, including nine original articles and one review, several predominant themes emerge. These include the enhancement and characterization of functional properties, the preservation of nutritional and bioactive profiles during processing and storage, the mitigation of microbial risks in fresh and minimally processed products, and the innovative use of fruit and vegetable co-products as feed additives. This latter approach not only contributes to reducing the environmental impact of food waste but also supports the sustainability of livestock production systems.

Notably, several studies explore innovative plant-based formulations with neuroprotective or anti-inflammatory potential, while others focus on the influence of cultivation conditions and post-harvest treatments on the accumulation of health-promoting compounds. The inclusion of research addressing shelf life and microbiological stability underlines the importance of aligning product innovation with consumer safety and industry requirements.

Collectively, this Special Issue highlights the necessity of a multidisciplinary approach to food innovation, one that integrates plant science, food technology, nutrition, and microbiology. We hope that the contributions presented here will inspire further research and development toward more sustainable, health-promoting, and safe plant-based food systems.

## Data Availability

Not applicable.
